# Bioinformatics prediction and experimental verification of a novel microRNA for myocardial fibrosis after myocardial infarction in rats

**DOI:** 10.7717/peerj.14851

**Published:** 2023-02-09

**Authors:** Qianqian Guo, Dandan Wu, Dongdong Jia, Xinyue Zhang, Aiming Wu, Lixia Lou, Mingjing Zhao, Mengzhu Zhao, Yijie Gao, Manman Wang, Menghua Liu, Meng Chen, Dongmei Zhang

**Affiliations:** 1 Key Laboratory of Chinese Internal Medicine of Ministry of Education and Beijing, Dongzhimen Hospital, Beijing University of Chinese Medicine, Beijing, China; 2 College of Traditional Chinese Medicine, Xinjiang Medical University, Urumqi, Xinjiang, China; 3 School of Traditional Chinese Medicine, Beijing University of Chinese Medicine, Beijing, China

**Keywords:** MicroRNA, Myocardial infarction, Myocardial fibrosis, Bioinformatics analysis, miR-199a-5p

## Abstract

**Background:**

MicroRNAs (miRNAs) are endogenous noncoding single-stranded small RNAs. Numerous studies have shown that miRNAs have pivotal roles in the occurrence and development of myocardial fibrosis (MF). However, miRNA expression profile in rats with MF after myocardial infarction (MI) is not well understood. The present study aimed to find the potential miRNA for MF post MI.

**Methods:**

SPF male Sprague-Dawley (SD) rat models of acute myocardial infarction (AMI) were established by ligating the anterior descending branch of the left coronary artery, while sham-operated rats were only threaded without ligation as a control group. Hematoxylin-eosin and Masson trichrome staining were used to detect myocardial histopathological changes for model evaluation. The differentially expressed miRNAs were detected by using the Agilent Rat miRNA gene chip in the myocardial tissue of the infarct marginal zone. Gene Ontology (GO) and Kyoto Encyclopedia of Genes and Genomes (KEGG) pathway enrichment analysis were performed by DAVID. The expression of miR-199a-5p was verified by real-time fluorescence quantitative PCR (qRT-PCR). Transfected miR-199a-5p mimics into cardiac fibroblasts (CFs) to construct cell models of miR-199a-5p overexpression. Dual-luciferase reporter assay was employed to validate the target gene of miR-199a-5p. The protein expression of the target gene in CFs transfected with miR-199a-5p mimics were detected by Western blot.

**Results:**

Myocardial fibrosis was exacerbated in the model group compared with the control group. Thirteen differentially expressed miRNAs between the two groups were screened and their expression levels in the model group were all higher than those in the control group. The expression of miR-199a-5p was significantly increased in the model group in qRT-PCR, which was consistent with the results of the gene chip. KEGG enrichment analysis showed that the target genes of miR-199a-5p were enriched in the insulin signaling pathway. Furthermore, dual-luciferase reporter assay indicated that miR-199a-5p could negatively regulate the expression of GSK-3β. After transfection, the expression of miR-199a-5p was increased in the miR-199a-5p mimics group. The protein expression of GSK-3β was decreased in CFs transfected with miR-199a-5p mimics.

**Conclusion:**

Our study identified miR-199a-5p could promote the progression of myocardial fibrosis after myocardial infarction by targeting GSK-3β, which provides novel targets for diagnosis and treatment of MF.

## Introduction

In recent years, a national survey of 1.7 million Chinese adults reported that approximately one in 10 adults had a high risk of cardiovascular disease (CVD) (Lu et al., 2019). Myocardial infarction (MI) has high morbidity and mortality; furthermore, severe myocardial ischemia and hypoxia inevitably cause necrotic cardiomyocytes to be replaced by collagen fibers, resulting in the formation of myocardial fibrosis (MF). In the initiation and progression of MF, cardiac fibroblasts (CFs) were activated by various cytokines, proliferate massively, and cause an imbalance between synthesis and degradation of extracellular matrices (ECM), leading to excessive deposit of ECM components, and finally result in arrhythmia, heart failure. However, the potential targets involved in MF post MI remain poorly understood (Wang, Lin & Bagchi, 2019).

MicroRNAs (miRNAs) are a class of highly conserved endogenous noncoding single-stranded small RNAs with a length of 18–22 nucleotides. They are involved in regulation at the posttranscriptional and translational levels through the degradation and translation inhibition of messenger RNA (mRNA). It is estimated that at least 60% of all mammalian genes may be regulated by miRNAs (Friedman et al., 2009). MiRNAs can help uncover the mechanisms of diseases and provide new entry points into therapy. Accumulated evidence has revealed that miRNAs are involved in the pathological process of CVD through specific signaling pathways (Wu et al., 2017). A study demonstrated that miR-199a deficiency can inhibit MF by targeting SFRP5 (Chen et al., 2019). Moreover, elevated expression of miR-199a-5p correlates with the progression of AMI (Yan et al., 2018). MiR-199a-5p, which is sensitive to hypoxia (Rane et al., 2009), was increased in oxygen-glucose deprivation and reperfusion (OGD/R)-treated H9c2 cells; furthermore, the miR-199a-5p mimics promoted OGD/R-treated cytotoxicity (Liu et al., 2019). Located on human chromosome 19 and ubiquitously expressed, miR-199a-5p is involved in multiple diseases, such as tumors and asthma (Li et al., 2019; Huang et al., 2018). Therefore, it is worth considering that miR-199a-5p is related to MF.

A growing number of studies have shown that energy metabolism imbalances such as glucose fluctuation can exacerbate cardiac remodeling and MF (Ying et al., 2017; Saito et al., 2014). Study has shown that heart failure after MI could lead to a shift from fatty acid oxidation to glucose oxidation (Cheng et al., 2020). To balance energy metabolism, the role of the insulin signaling pathway has become increasingly highlighted. In response to elevated levels of blood glucose, insulin promptly binds to receptors. The insulin receptor substrate (IRS) protein, a key mediator of the insulin signaling pathway, is required for hormonal control of metabolism (Copps & White, 2012). After phosphorylation, IRS initiates a cascade of downstream signaling, such as activation of the phosphatidylinositol 3-kinase (PI3K)/Protein kinase B (AKT) pathway. A prior study showed that insulin-like growth factor-1 (IGF-1) may protect cardiac tissue against myocardial ischemia/reperfusion (I/R) injury through the PI3K/Akt pathway in rats (Liao et al., 2019). PI3K/Akt signaling pathway can phosphorylate GSK-3β and lead to its inactivation (Liang et al., 2015). GSK-3β in active state can effectively inhibit fibrosis and is a vital regulator of fibrosis (Zheng et al., 2020). Lal et al. (2014) have shown that GSK-3β knockout mice induce the transformation of fibroblasts to myofibroblasts, resulting in ECM deposition and fibrosis formation, suggesting that GSK-3β is a crucial inhibitor of myocardial fibrosis in cardiac remodeling.

**Figure 1:**
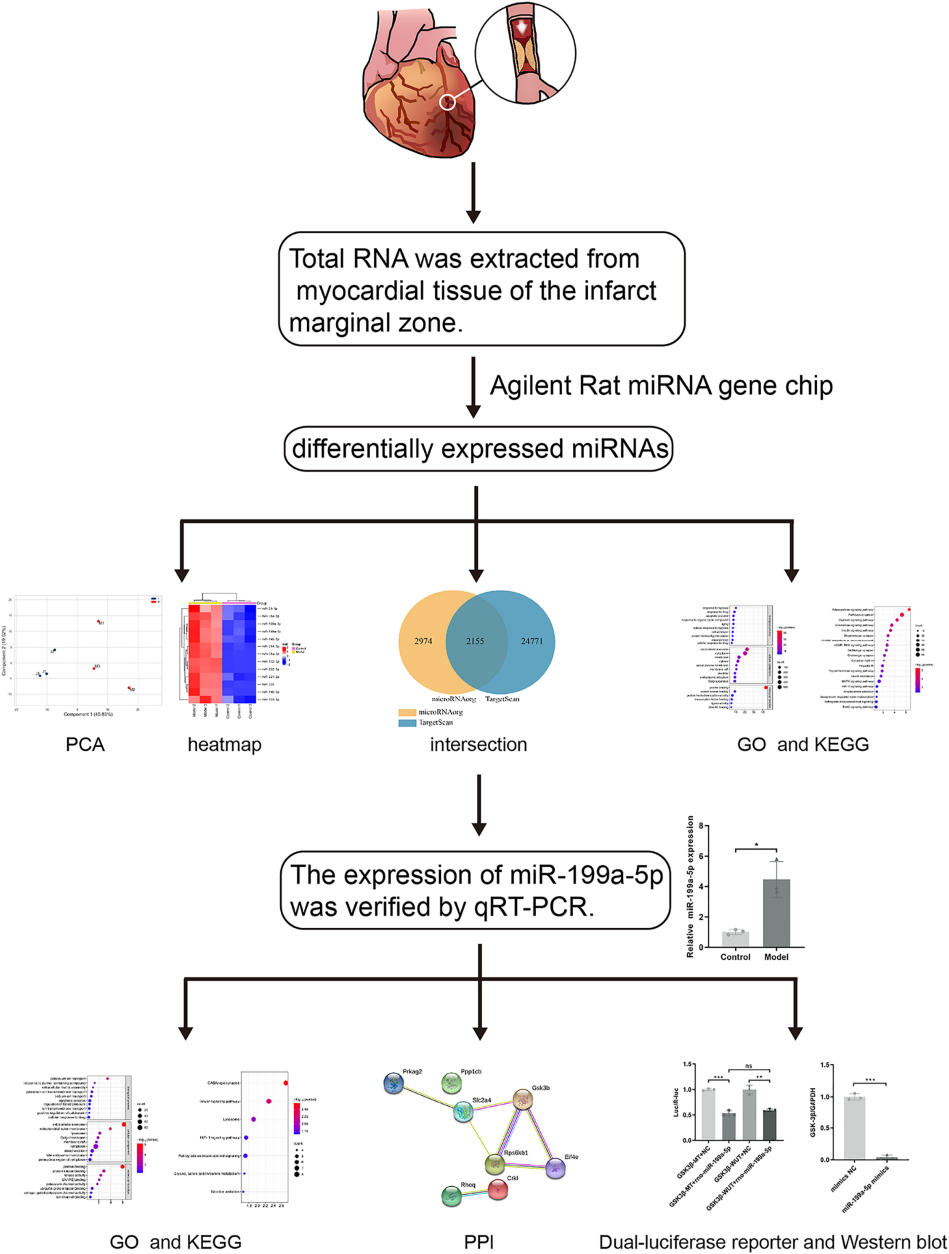
The workflow of this study. Rat models of acute myocardial infarction (AMI) were established by ligating the anterior descending branch of the left coronary artery. Total RNA was extracted from myocardial tissue of the infarct marginal zone. The differentially expressed miRNAs were detected by using the Agilent Rat miRNA gene chip. Gene Ontology (GO) and Kyoto Encyclopedia of Genes and Genomes (KEGG) pathway enrichment analysis were performed by DAVID. The expression of miR-199a-5p was verified by real-time fluorescence quantitative PCR (qRT-PCR). KEGG enrichment analysis showed that the target genes of miR-199a-5p were enriched in the insulin signaling pathway. Furthermore, dual-luciferase reporter assay indicated that miR-199a-5p could negatively regulate the expression of GSK-3β. The protein expression of GSK-3β was decreased in CFs transfected with miR-199a-5p mimics.

Previous studies have found that MI in rats can cause the accumulation of collagen-based ECM, and lead to MF, however, the potential targets are not fully understood. With the development of the microarray technology and bioinformatics techniques, the disease-related miRNAs have been discovered. Therefore, in this study (Fig. 1), rat models of MF after MI were established. MiRNA expression profile was detected by using the Agilent Rat miRNA gene chip and bioinformatics analysis was performed by DAVID. The expression of miR-199a-5p was verified by qRT-PCR. Dual-luciferase reporter assay was designed to validate the relationship between miR-199a-5p and its target gene. To find the potential targets for diagnosis and treatment of MF post MI.

## Materials and Methods

### Establishment of experimental animal models

**Figure 2:**
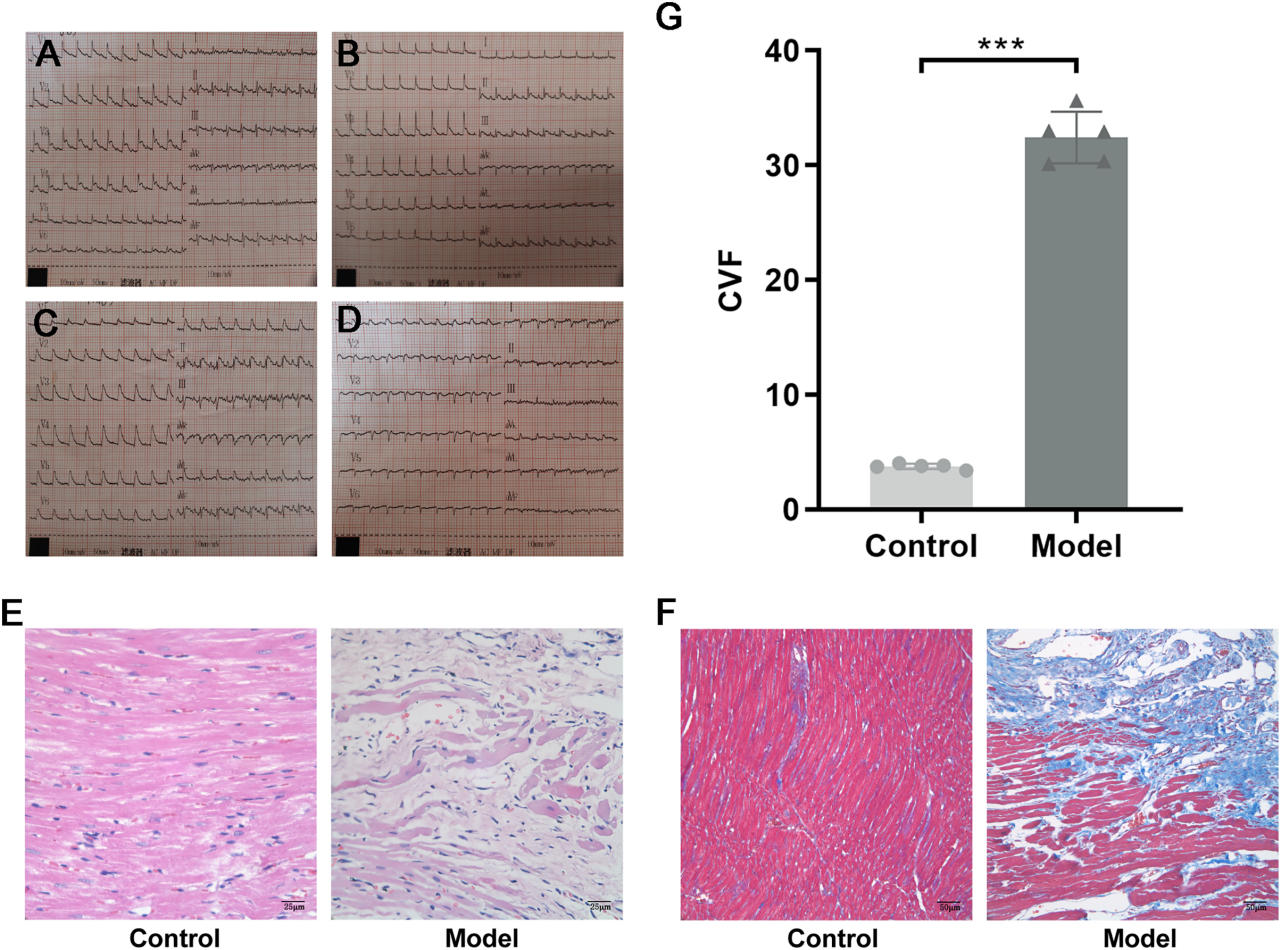
Evidence of successful LAD ligation. (A) Immediate postoperative electrocardiogram of rats in control group. (B) Electrocardiogram of rats in control group 24 h after operation. (C) Immediate postoperative electrocardiogram of rats in model group. (D) Electrocardiogram of rats in model group 24 h after operation. (E) Representative images of hematoxylin-eosin (HE) staining of myocardial tissue in the control and model groups. (F) Representative images of Masson trichrome staining of myocardial tissue in the control and model groups. (G) CVF in myocardial tissue in the control and model groups. *n* = 5, ***P < 0.0001, t = 28.21, df = 8 compared with the control group.

SPF male Sprague–Dawley (SD) rats weighing 200 ± 20 g were purchased from Beijing Vital River Laboratory Animal Technology Co. Ltd., Raised in the Barrier Animal Laboratory of Dongzhimen Hospital, Beijing University of Chinese Medicine, with a 12 h light/dark cycle and *ad libitum* food and water. All protocols and applications of animals in this study were approved by The Animal Care & Welfare Committee of Dongzhimen Hospital, Beijing University of Chinese Medicine (No: 16–25). The rats were divided into model group and sham-operated group by random number table. A model of AMI was established by ligating the left anterior descending coronary artery (LAD) of the rat heart. The rat was anesthetized intraperitoneally with 1% sodium pentobarbital (40 mg/kg). The skin was cut laterally at the third and fourth ribs of the left chest, approximately 1.5 cm in length. The musculature was bluntly separated, the rib was braced with a thoracotomy and the thymus was fixed upward to fully enlarge the surgical field, and the pericardium was carefully torn open to expose the ligation site of the rat heart. The anterior descending branch of the left coronary artery was ligated with a 5/0 line approximately 2 mm below the left atrial appendage and the arterial cone. The anterior myocardial wall was quickly ischemic and whitening, and the electrocardiogram (ECG) revealed saddle-back-like ST-segment elevations. After 24 h, the ECG was conducted again, and pathological Q waves appeared in the chest leads and lead I and aVL, indicating that the model was successful (Fig. 2A). The sham-operated rats were only threaded without ligation as a control group. There were five rats in each group and ten rats in total. The rats were fed adequate food and deionized water for 4 successive weeks. Finally, the rats were euthanized with 1% sodium pentobarbital (40 mg/kg), and myocardial tissue was collected at the infarct margin.

### Histopathological staining

Dehydrated, paraffin-embedded myocardial tissues were cut into 3-μm-thick sections. The sections were dewaxed in xylene and rehydrated stepwise in a descending ethanol series. For hematoxylin-eosin (HE) staining, the sections were stained with hematoxylin staining solution and eosin staining solution (BLB-03 and YH-102, Beijing Kyushu Berlin Biotechnology Co. Ltd., Beijing, China). For Masson trichrome staining, the sections were dyed with nuclear staining, cytoplasmic staining, color separation solution, and counterstaining solution (D026-20, Nanjing Jiancheng Bioengineering Institute, Jiangsu, China) in sequence. The samples were dehydrated with ethanol in a series of concentrations and cleared with xylene. Coverslips were mounted with neutral balsam and sections were photographed by light microscopy. With the help of Image-Pro Plus 6.0, the area of myocardial collagen could be quantified. Collagen volume fraction (CVF) = Collagen area/(Collagen area + Myocardial area) × 100%. Five nonoverlapping fields were randomly selected for each section (×200), and the results are presented as the mean.

### MiRNA gene chip

Due to the cost of microarray, three myocardial tissue samples were randomly selected from each group for microarray detection. Total RNA was extracted from myocardial tissue according to the mirVanaTM RNA Isolation Kit (AM1561, Applied Biosystems, Waltham, MA, USA). The Agilent Rat miRNA gene chip was used in this research. The hybridization and scanning of the miRNA gene chip were carried out. The standardized data were filtered, and at least one set of 100% probes marked as “Detected” in the two sets of samples used for comparison was left for subsequent analysis. The fold change value and *p* value (*FC* > 2, *P* < 0.05) based on a t test were used to screen differentially expressed miRNAs. The differentially expressed miRNAs were clustered in an unsupervised hierarchy, and the expression patterns of the differentially expressed miRNAs among different samples were displayed in the form of heatmaps. The microarray data discussed in this study have been deposited in the National Center for Biotechnology Information (NCBI) Gene Expression Omnibus (GEO) and are accessible through (GEO) Series accession number GSE208159.

### MiRNAs bioinformatics analysis

TargetScan and microRNAorg databases were used to predict target genes of differentially expressed miRNAs, and the intersection was taken for subsequent analysis. Database for Annotation, Visualization and Integrated Discovery (DAVID) (https://david.ncifcrf.gov/) is a common software for target genes. The Gene Ontology (GO) and Kyoto Encyclopedia of Genes and Genomes (KEGG) databases were used to perform enrichment analyses to elucidate biological functions and pathways regulated by miRNAs (*P* < 0.05). The protein-protein interaction (PPI) network was constructed using the STRING database and Cytoscape software.

### MiRNAs expression analysis

Total RNA was extracted from myocardial tissue using the mirVanaTM RNA Isolation Kit (AM1561; Applied Biosystems, Waltham, MA, USA). The RNA to be tested was reverse transcribed into cDNA using the miScript II Reverse Transcription Kit (Qiagen, Hilden, Germany). Using cDNA as a template, real-time PCR was performed with QuantiFast® SYBR® Green PCR Master Mix (Qiagen, Hilden, Germany). Reactions were incubated at 95 °C for 5 min, followed by 40 cycles of 95 °C for 10 s and 60 °C for 30 s. The expression levels of microRNAs were normalized to U6 and calculated using the 2^−ΔΔCT^ method. The microRNA-specific primer sequences were as follows: sense CCAGTGTTCAGACTACCTGTTC for rno-miR-199a-5p, sense CAAGGATGACACGCAAATTCG for U6.

### Extraction and culture of primary cardiac fibroblasts

The 3-day-old Wistar suckling rats were purchased from Beijing Vital River Laboratory Animal Technology Co. Ltd. All protocols and applications of animals in this study were approved by The Animal Care & Welfare Committee of Dongzhimen Hospital, Beijing University of Chinese Medicine (No: 21–06). The 3-day-old Wistar suckling rats were sacrifice by cervical dislocation and disinfected with alcohol three times. The skin on the chest was exposed, and cut perpendicular to the ribs along the left margin of the breastbone of the infant rats at 0.5 cm with sterile ophthalmic straight scissors. The heart was extruded by gently squeezing the incision, and the apex of heart was cut off with a sterile ophthalmic curved scissors and quickly placed in precooled PBS (SH30256.01, Hyclone, Logan, UT, USA). Using the Neonatal Heart Dissociation Kit (130-098-373; Miltenyi Biotechnology, Bergisch Gladbach, Germany), the myocardial tissue was digested under aseptic conditions. The digested tissue was seeded in culture flasks with DMEM (C11995500BT; Gibco, WA, Australia) containing 15% fetal bovine serum (FBS) (10099141; Gibco, Hackett Drive Crawley, WA, Australia), 1% penicillin, and streptomycin in an incubator at 37 °C and 5% CO_2_. After 90 min, the unattached cells were discarded, and the medium was replaced with 10% FBS-DMEM. The experiments used 3–5 generations of cardiac fibroblasts (CFs).

### Immunocytochemistry

CFs were inoculated on 24-well polylysine-coated sterile slides with 8 × 10^4^ CFs per well. After 24 h incubation at 37 °C and 5% CO_2_ incubator, CFs were sequentially fixed with 4% paraformaldehyde, permeated with 0.5% triton and inactivated endogenous peroxidase with 3% H_2_O_2_. CFs were incubated with anti-Vimentin (60330-1-Ig, proteintech) and anti-alpha-smooth muscle actin (α-SMA) (55135-1-AP, proteintech) antibodies overnight at 4 °C. After washing with PBS, the CFs were incubated with a secondary antibody. DAB chromogenic reagent was added in the dark environment, claybank was positive expression, and no chromogenic was negative expression. Hematoxylin restained the nucleus.

### MiRNA transfection

CFs were digested and inoculated into culture plates. After 24 h, DMEM without FBS, growth factor, penicillin, and streptomycin was replaced and transfected with miR-199a-5p mimics (GenePharma, Suzhou, China). The CFs were cultured in an incubator at 37 °C and 5% CO_2_ for 6 h and then the medium was replaced with 10% FBS-DMEM.

### Dual-luciferase reporter assay

293T cells were co-transfected with rno-miR-199a-5p mimic or miR-NC and pMIR-REPORT Luciferase-GSK3β 3′UTR (WT) or pMIR-REPORT luciferase-GSK3β3′UTR (MUT), respectively. A total of 48 h after transfection, Luciferase activity was detected by microplate reader.

### Western blot

CFs was homogenized with RIPA lysis buffer containing protease and phosphatase inhibitors. After SDS–PAGE, the proteins were transferred to polyvinylidene difluoride (PVDF) membranes. The membranes were blocked with 5% skim milk powder. The PVDF membranes were incubated with anti-GSK-3β (12456, CST) and anti-GAPDH (ab8245; Abcam, Cambridge, UK) antibodies overnight at 4 °C. After washing with Tris-buffered saline with 0.05% Tween-20 (TBST), the membranes were incubated with a secondary antibody. Membranes were placed in an enhanced chemiluminescence (ECL) mixture and then exposed, developed, and fixed in a dark room. Using GAPDH as the internal reference, the immunoreactive bands were analyzed using ImageJ analysis software.

### Statistical analysis

The data are presented as the mean ± standard deviation (SD). Statistical analysis was performed with SPSS 20.0 and GraphPad Prism 8. When the data were normally distributed, an unpaired t test was conducted for comparisons between two groups. The nonparametric Kruskal–Wallis test was used when the data did not obey a normal distribution. Statistical significance was defined when *P* < 0.05.

## Results

### Evidence of successful LAD ligation

HE staining showed that the structure of cardiomyocytes in the control group was complete, with uniform morphology, clear nuclei, a uniformly stained cytoplasm, and tightly arranged muscle bundles. However, in the model group, the myocardial tissue structure was destroyed, with a large area of cardiomyocyte fragmentation, nuclear pyrosis and deformation, disordered arrangement of muscle fibers, and widening of space (Fig. 2B). To investigate the collagen content, Masson trichrome staining was used. Compared with the control group, a large amount of collagen was interspersed between cardiomyocytes, and CVF was significantly increased in the model group (*P* < 0.0001, t = 28.21, df = 8, Figs. 2C and 2D). These results suggested that in rats with MI, a large number of necrotic cardiomyocytes were replaced by collagen, indicating that MF was formed.

### MiRNAs expression profile and bioinformatics analysis

**Figure 3:**
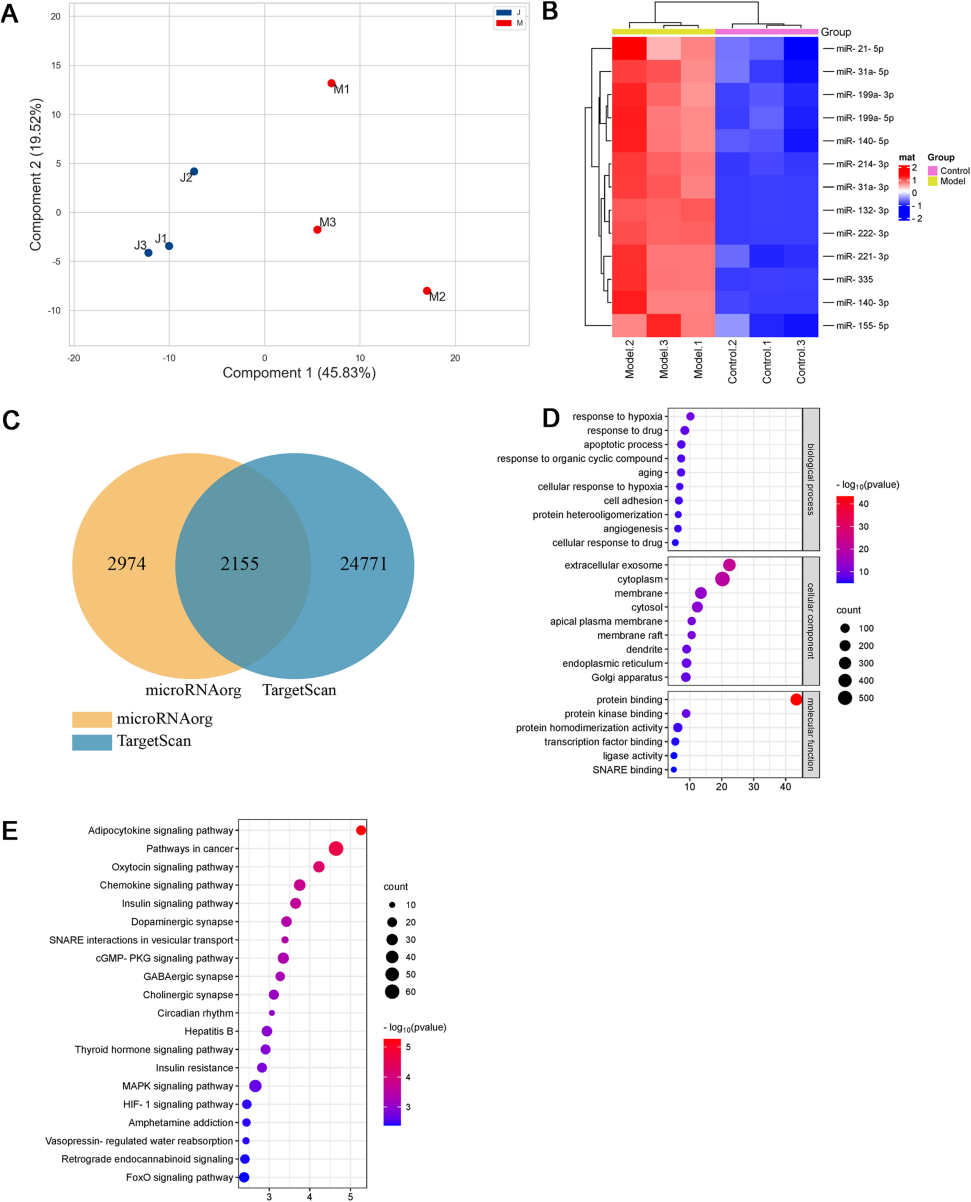
Differential miRNAs and bioinformatics analysis. (A) PCA plot of control (J) and model (M) rats. (B) Heatmap of differentially expressed miRNAs in the myocardial tissue of the infarct marginal zone. The color scale indicates the change in gene expression from relatively low (blue) to relatively high (red). (C) The intersection diagram of target genes of differentially expressed miRNAs. (D) GO analysis of target genes of differentially expressed miRNAs. The vertical axis represents GO function items. (E) KEGG signaling pathway analysis of target genes of differentially expressed miRNAs. The vertical axis represents the KEGG signaling pathway, and the horizontal axis represents the Gene Ratio, that is, the ratio of the number of genes in a KEGG pathway to the number of all the annotated genes in the KEGG pathway. The larger the proportion of genes, the higher the degree of enrichment.

As shown in Figs. 3A and 3B and Table 1, thirteen differentially expressed miRNAs were screened using the Agilent Rat miRNA gene chip (*FC* > 2, *P* < 0.05), and their expression levels in the model group were higher than those in the control group. In the longitudinal sample clustering tree, the model group was clustered into one cluster, while the control group was clustered into another cluster, indicating that the expression levels of miRNAs between the two groups had prominent differences. In the horizontal sample clustering tree, miRNAs with similar biological functions were clustered. Red indicates that the expression of miRNA was upregulated, and blue indicates that the expression of miRNA was downregulated; the brighter the color, the greater the difference.

**Table 1:** Differentially expressed miRNAs between model group and control group.

miRNAs	*FC*	*P* value	Regulation (Model *vs* Control)
rno-miR-132-3p	35.291122	0.000000105	Up
rno-miR-140-3p	2.527923	0.00046195	Up
rno-miR-140-5p	2.5984645	0.000968062	Up
rno-miR-155-5p	2.156454	0.002853242	Up
rno-miR-199a-3p	2.9326804	0.000630609	Up
rno-miR-199a-5p	4.010446	0.000921005	Up
rno-miR-21-5p	2.046981	0.006777289	Up
rno-miR-214-3p	3.2471766	0.000057200	Up
rno-miR-221-3p	2.234359	0.00043476	Up
rno-miR-222-3p	112.03117	0.000001474	Up
rno-miR-31a-3p	77.02281	0.000066152	Up
rno-miR-31a-5p	10.639086	0.000772346	Up
rno-miR-335	22.00288	0.000119804	Up

**Note:**

*FC* > 2, *P* < 0.05.

MiRNAs, small single-stranded noncoding RNAs (ncRNAs), can regulate the expression of target genes. In this study, two databases, microRNAorg and TargetScan, were used to predict target genes of the thirteen differentially expressed miRNAs, and 2155 common target genes were obtained (Fig. 3C). Through GO analysis of common target genes, the function of differential miRNAs could be described. As shown in Fig. 3D, GO included three parts: cellular component, molecular function, and biological process. Enrichment statistics were performed on the target genes in each GO entry (*P* < 0.01). KEGG enrichment analysis of common target genes was performed to predict the signaling pathways that differentially expressed miRNAs might target. As shown in Fig. 3E, 63 signaling pathways targeted by differentially expressed miRNAs were obtained, including the adipocytokine signaling pathway, chemokine signaling pathway, insulin signaling pathway, insulin resistance, MAPK signaling pathway, HIF-1 signaling pathway, TNF signaling pathway, and PPAR signaling pathway (*P* < 0.05).

### MiRNA expression

**Figure 4:**
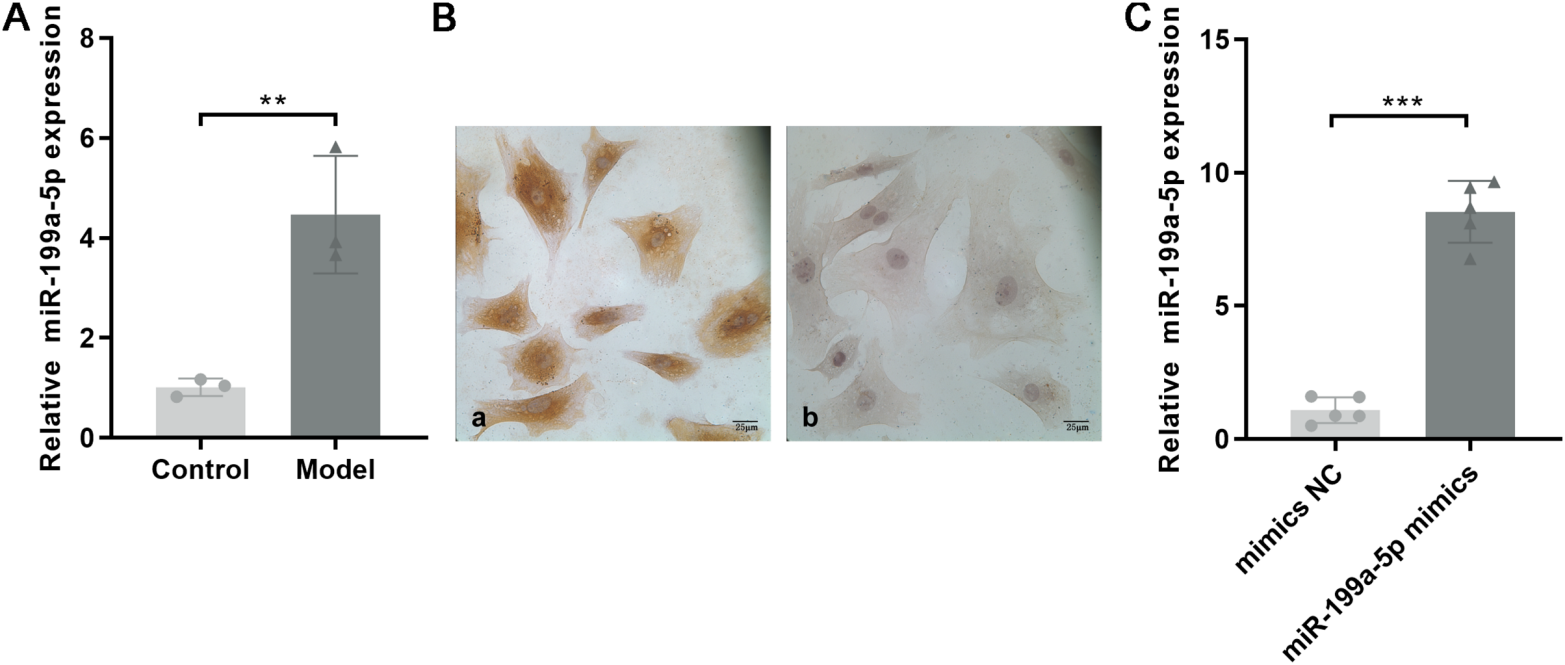
Expression of miR-199a-5p. (A) Relative expression of miR-199a-5p in control and model rats. *n* = 3, **P = 0.01, t = 5.033, df = 4 compared with the control group. (B) CFs were identified by immunohistochemical staining. (a) Anti-vimentin antibody staining; (b) anti-α-SMA antibody staining. Scale bar: 25 μm. (C) The expression of miR-199a-5p in CFs transfected with miR-199a-5p mimics. *n* = 5, ***P < 0.0001, t = 13.22, df = 8 compared with the mimics NC group.

Based on the miRNA expression profile, we selected miR-199a-5p that showed significantly upregulated expression for qRT-PCR validation in the myocardial tissue with MF post MI. As shown in Fig. 4A, the expression of miR-199a-5p in the model group was increased, which was consistent with the results of the gene chip (*P* = 0.01, t = 5.033, df = 4).

CFs were extracted and identified by immunocytochemistry. Vimentin is commonly used to characterize the cells of fibroblast origin, while α-SMA is used to recognize myofibroblasts (Tarbit et al., 2019). Immunohistochemical staining showed that Vimentin, located in the cell cytoplasm, was positively expressed in CFs, and α-SMA was negatively expressed (Fig. 4B).

To further confirm the role of miR-199a-5p in MF, miR-199a-5p mimics were transfected into CFs. After transfection, the expression level of miR-199a-5p was increased in the miR-199a-5p mimics group, indicating that the transfections were successful (*P* < 0.0001, t = 13.22, df = 8, Fig. 4C). These results suggested that miR-199a-5p was activated and played a crucial role in the progression of MF.

### Bioinformatics analysis of miR-199a-5p

**Figure 5:**
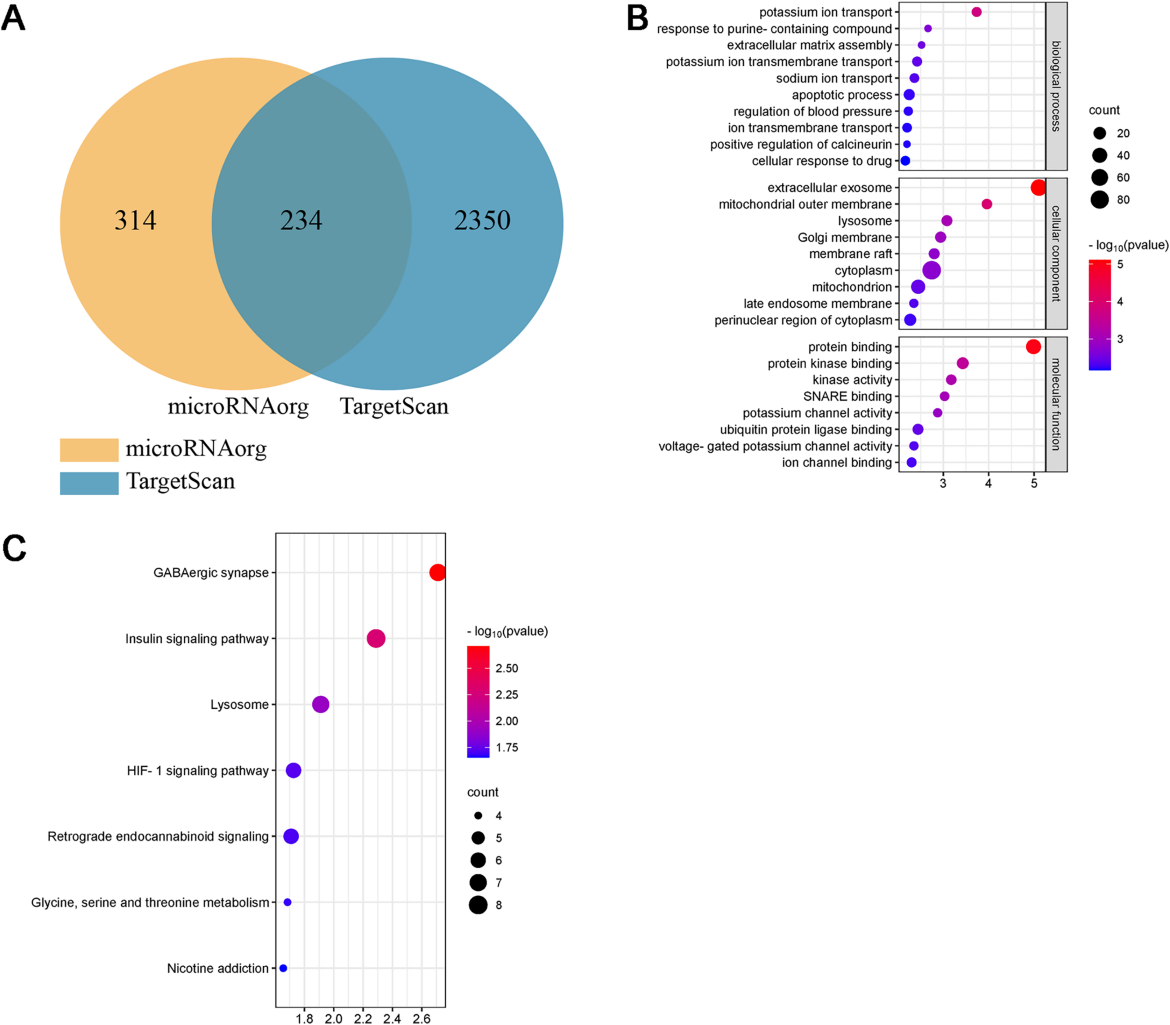
Bioinformatics analysis of miR-199a-5p. (A) The intersection diagram of target genes of miR-199a-5p. (B) GO analysis of target genes of miR-199a-5p. The vertical axis represents GO function items, and the horizontal axis represents the three parts of GO: biological process, cellular component, and molecular function. (C) KEGG signaling pathway analysis of target genes of miR-199a-5p. The vertical axis represents the KEGG signaling pathway, and the horizontal axis represents the Gene Ratio, that is, the ratio of the number of genes in a KEGG pathway to the number of all the annotated genes in the KEGG pathway. The larger the proportion of genes, the higher the degree of enrichment.

Studies have shown that the miR-199a family is closely related to CVD, suggesting that miR-199a-5p may play an important role in MF after MI. To further evaluate the biological function of miR-199a-5p, target genes prediction were performed using the microRNAorg and TargetScan databases, and 234 identical target genes were obtained (Fig. 5A). As shown in Fig. 5B, GO analysis included three parts: cellular component, molecular function, and biological process. Enrichment statistics were performed on the target genes in each GO entry (*P* < 0.01). KEGG analysis of miR-199a-5p target genes showed that a total of 7 signaling pathways (*FC* > 2, *P* < 0.05, Fig. 5C) were screened, such as GABAergic synapse, insulin signaling pathway, Lysosome, HIF-1 signaling pathway, retrograde endocannabinoid signaling, glycine, serine and threonine metabolism, nicotine addiction.

Interestingly, we found that insulin signaling pathway was prominent not only in the KEGG enrichment analysis of 13 miRNAs target genes, but also in the KEGG enrichment analysis of miR-199a-5p target genes. Therefore, insulin signaling pathway became the object of our study.

### Target gene of miR-199a-5p

**Figure 6:**
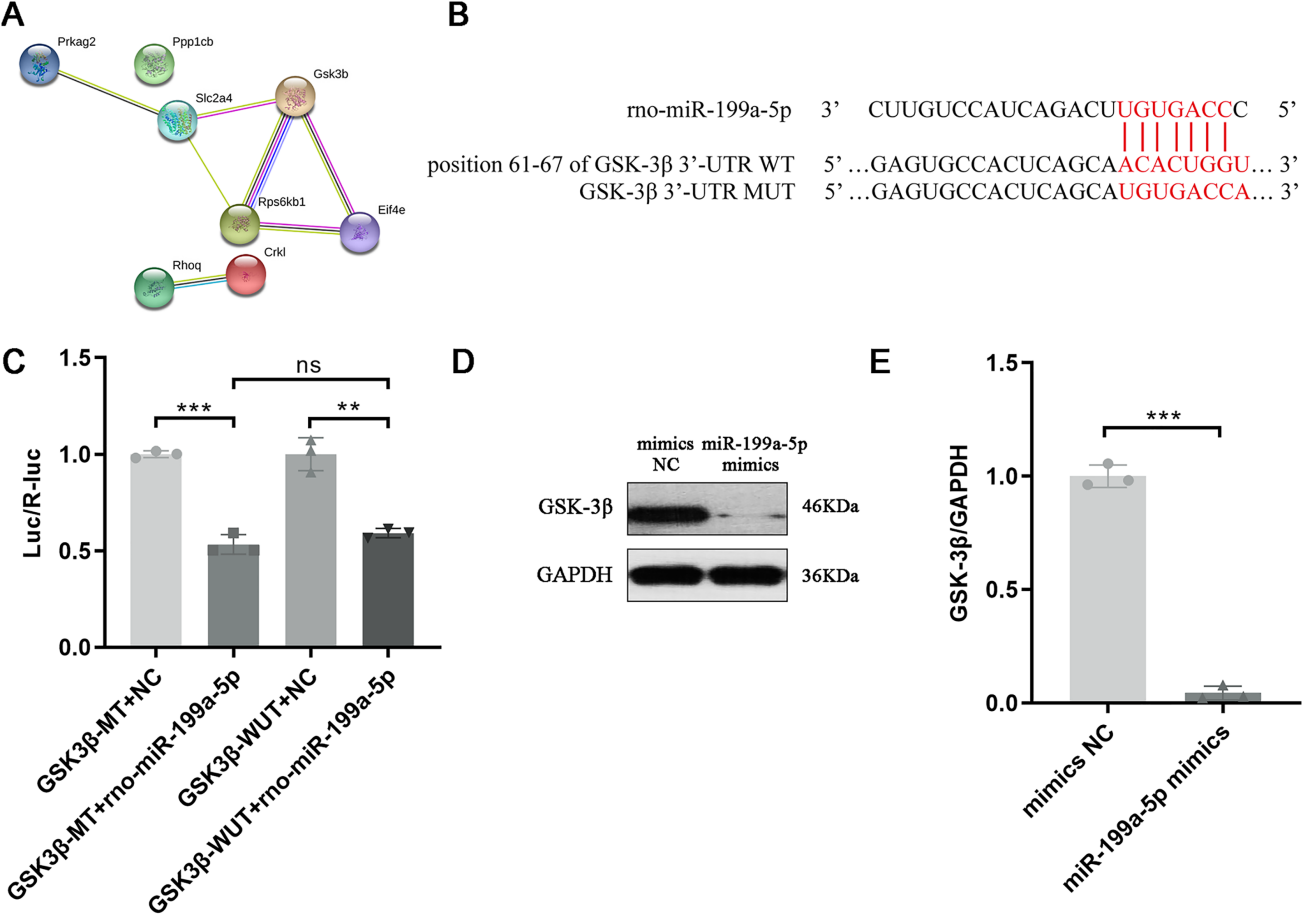
Target gene of miR-199a-5p. (A) The PPI network of proteins in the insulin signaling pathway. (B) The binding site between rno-miR-199a-5p and GSK-3β. (C) Dual-luciferase reporter assay, ***P = 0.0001, t = 15.01, df = 4 compared with the GSK3β-WT+NC group or **P = 0.0013, t = 8.03, df = 4 compared with the GSK3β-MUT+NC group. (D, E) The protein expression of GSK-3β in CFs transfected with miR-199a-5p mimics. *n* = 3, ***P < 0.0001, t = 28.72, df = 4 compared with the mimics NC group.

Above results indicated that miR-199a-5p could regulate multiple functions and signaling pathways through multiple target genes and suggested that miR-199a-5p might be involved in the process of MF after MI through the insulin signaling pathway. Although there are many target genes of miR-199a-5p, KEGG analysis showed that only eight target genes of miR-199a-5p were clustered in the insulin signaling pathway, including Ppp1cb, Gsk3β, Slc2a4, Prkag2, Rps6kb1, Rhoq, Crkl, and Eif4e. We performed PPI analysis on these target genes, the results showed that GSK-3β interacted with multiple proteins, such as Slc2a4, Rps6kb1, and Eif4e, which was of great significance in the insulin signaling pathway (Table 2, Fig. 6A). As shown in Fig. 6B, the binding site between rno-miR-199a-5p and GSK-3β was predicted by bioinformatics website (http://www.mirdb.org). By Dual-luciferase reporter assay, we indicated that overexpression of rno-miR-199a-5p inhibited the luciferase activity of wild-type GSK-3β 3′-UTR, but it also suppress luciferase activity of mutated one (*P* = 0.0001, t = 15.01, df = 4 or *P* = 0.0013, t = 8.03, df = 4, Fig. 6C). The result indicated that rno-miR-199a-5p could inhibit the expression of GSK-3β, but not by the predicted binding site. Rno-miR-199a-5p also regulated the luciferase expression of mutated GSK-3β 3′-UTR, it may be because in addition to the mutated binding sites, there may be other atypical binding sites or the overall downregulation of target gene expression may be caused by the indirect effect of overexpression of rno-miR-199a-5p. Furthermore, the protein expression of GSK-3β was decreased in CFs transfected with miR-199a-5p mimics (*P* < 0.0001, t = 28.72, df = 4, Figs. 6D and 6E).

**Table 2:** The KEGG enrichment analysis of miR-199a-5p target genes.

ID	Description	*P* value	Genes
rno04727	GABAergic synapse	0.0019461	Cacna1b, Slc6a1, Gabrb2, Gng5, Nsf, Gabrb3, Adcy3
rno04910	Insulin signaling pathway	0.0051442	Prkag2, Eif4e, Rhoq, Rps6kb1, Gsk3b, Ppp1cb, Crkl, Slc2a4
rno04142	Lysosome	0.0122634	Cd164, Atp6ap1, Manba, Atp6v0c, Lgmn, Dnase2b, Gm2a
rno04066	HIF-1 signaling pathway	0.0188008	Eif4e, Hif1a, Rps6kb1, Pdha1, Cdkn1b, Epo
rno04723	Retrograde endocannabinoid signaling	0.0195286	Cacna1b, Gabrb2, Gng5, Slc17a6, Gabrb3, Adcy3
rno00260	Glycine, serine and threonine metabolism	0.020662	Srr, Pipox, Agxt2, Dmgdh
rno05033	Nicotine addiction	0.0220968	Cacna1b, Gabrb2, Slc17a6, Gabrb3

## Discussion

A report from the American Heart Association updated in 2022 showed that in 2019, 27% of the world’s deaths were due to CVD, making it the leading cause of death globally, furthermore, in 2020, 19.05 million deaths were estimated for CVD globally, an increase of 18.71% compared to 2010 (Tsao et al., 2022). In CVD, MI is a leading cause of morbidity and mortality, and MF seriously affects the prognosis of patients. In this study, a model of AMI was established by ligating the left anterior descending coronary artery of rats and observing them for 4 weeks. The histopathological staining results showed that a large number of necrotic cardiomyocytes were replaced by collagen, leading to fibrosis formation in the model group.

To find the potential miRNAs involved in MF after MI, the Agilent Rat miRNA gene chip was used to screen the differentially expressed miRNAs between the two groups. Results showed that miR-199a-5p was up-regulated in the model group, which was verified by qRT-PCR. A prior study indicated that members of the miR-199a family, namely, miR-199a-3p and miR-199a-5p, are potential regulators of cardiac homeostasis (Joris et al., 2018). The expression of miR-199a-5p was markedly increased in cardiac hypertrophy in response to different stimuli *in vivo* and *in vitro* (Zhang et al., 2016). Therefore, it is worth considering that miR-199a-5p may have an important effect on the occurrence and development of MF. Studies have indicated that miRNAs regulate multiple biological processes by directly binding to target genes (Bartel, 2004). Furthermore, each miRNA can regulate the expression of multiple mRNAs (Peter, 2010). Target genes prediction of miR-199a-5p were performed using the microRNAorg and TargetScan databases, and 234 identical target genes were obtained. Furthermore, dual-luciferase reporter assay indicated that miR-199a-5p could negatively regulate the expression of GSK-3β. Glycogen synthase kinase-3 (GSK-3) is a multifunctional serine/threonine-protein kinase composed of two subtypes (α and β) that are encoded by different genes and ubiquitously expressed. Numerous studies have demonstrated that GSK-3β, but not GSK-3α, has cardioprotective effects (Mokhtari et al., 2015; Zuo et al., 2016). GSK-3β is activated in resting cells. Studies have confirmed that overexpression of GSK-3β has a crucial effect on myocardial hypertrophy, including the suppression of protein synthesis and the expression of hypertrophic genes, while inhibition of GSK-3β can lead to aggravation of myocardial hypertrophy (Takahashi-Yanaga, 2018).

MiRNAs have been reported to regulate CVD through various mechanisms, such as the TGF-β signaling pathway (Zhao et al., 2018) and the PI3K/Akt signaling pathway. In this study, KEGG enrichment analysis showed that the target genes of miR-199a-5p were enriched in the insulin signaling pathway. The insulin signaling pathway is a common signaling pathway to regulate myocardial energy metabolism. The binding of insulin to the corresponding receptor can phosphorylate insulin substrate, thus activating PI3K/Akt signaling pathway (Ye et al., 2022). The phosphorylation of Akt leads to the phosphorylation of many downstream targets, such as GSK-3β, mediating the cell growth and proliferation and glucose metabolism regulation (Plotnikov et al., 2011). There are severe systemic and myocardial glucose metabolism disorders in T2DM patients complicated with heart failure, resulting in significant abnormalities of myocardial energy metabolism and dysfunction of myocardial contraction. The insulin signaling pathway can improve abnormal energy metabolism in the whole body and myocardium, in which the core is the activation of Akt (Ye et al., 2022). However, over-expression of Akt can cause pathological myocardial hypertrophy (Dorn & Force, 2005). As a potential risk factor, excessive activation of insulin signaling pathway may worsen MF.

In summary, a novel miRNA, miR-199a-5p, was confirmed to be involved in the pathological process of MF post MI. However, the present study has several limitations. First of all, in this experiment, SD rats were used for surgery procedures, and Wistar suckling rats were used for fibroblast isolation. The design of this study was not rigorous enough because the same strain was not selected for the experiment. Currently, Wistar rats and SD rats are commonly used closed group rats. Although SD rats are bred from Wistar rats, they are not identical, which will be noted in future experiments. But it is worth mentioning that both of them can be used to prepare MF models. Secondly, there was left unblinded due to the nature of the analysis being carried out. Finally, due to the small amount of myocardial tissue in the control group and model group in this experiment, it was not enough for Western blot test to detect the expression of GSK-3β protein. In the future, we will build more animal models and reserve enough myocardial tissue for Western blot detection to show the expression of GSK-3β protein. Furthermore, multiple miRNAs and a complex network of signaling pathways are involved in the occurrence and development of MF. The pathogenesis is complex, and more elaborate mechanisms need to be elucidated in future studies.

## Conclusions

In conclusion, our study identified miR-199a-5p could promote the progression of myocardial fibrosis after myocardial infarction by targeting GSK-3β, which plays an important role in the insulin signaling pathway, which provides novel targets for diagnosis and treatment of MF.

## Supplemental Information

10.7717/peerj.14851/supp-1 Supplemental Information 1 Raw data.

10.7717/peerj.14851/supp-2 Supplemental Information 2 Full Author Checklist.

10.7717/peerj.14851/supp-3 Supplemental Information 3 Gels and films in Figure 6D.

## Additional Information and Declarations

### Microarray Data Deposition

The following information was supplied regarding the deposition of microarray data: The microarray data are available at NCBI GEO: GSE208159.

### Data Availability

The following information was supplied regarding data availability: The raw data in this study are available in the Supplemental File.
