# Supplementary figures and images for: Bioinformatics prediction and experimental verification of a novel microRNA for myocardial fibrosis after myocardial infarction in rats
Source: PeerJ. 2023 Feb 9;11:e14851. doi: 10.7717/peerj.14851 (PMC9922498; doi:10.7717/peerj.14851)

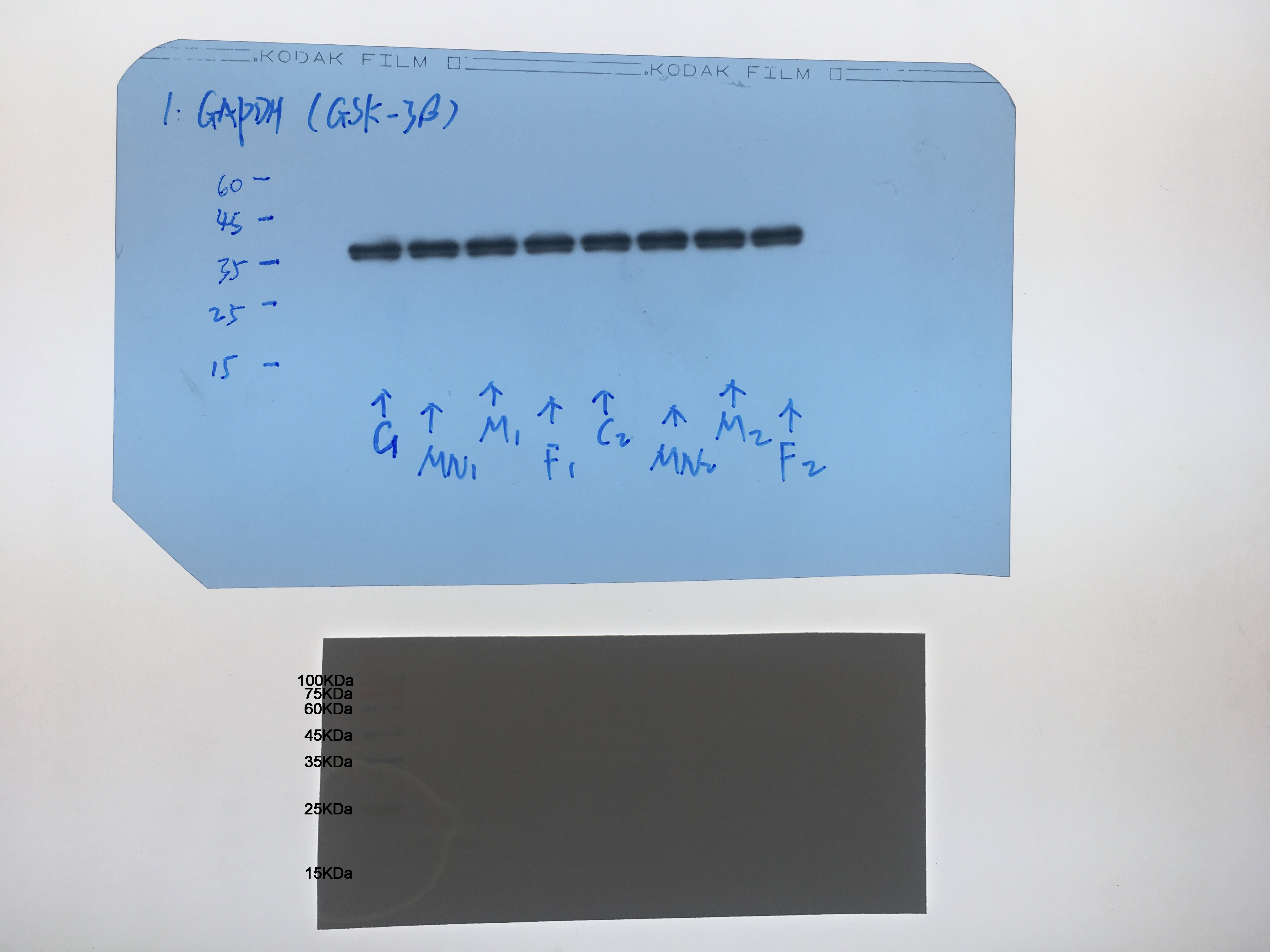

Supplement: Supplemental Information 3 [file peerj-11-14851-s003.zip › Figure6D-GAPDH-1.jpg]

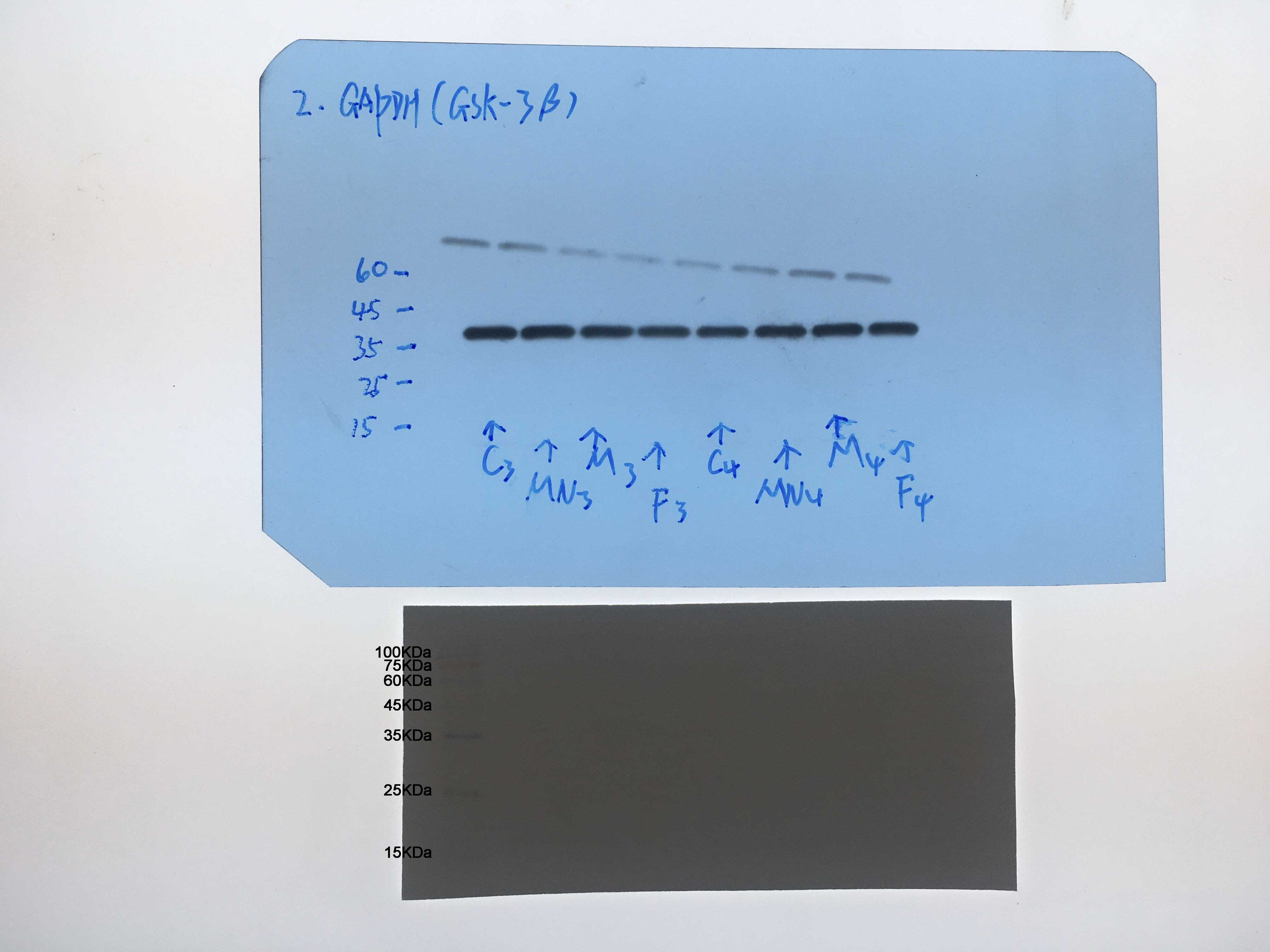

Supplement: Supplemental Information 3 [file peerj-11-14851-s003.zip › Figure6D-GAPDH-2.jpg]

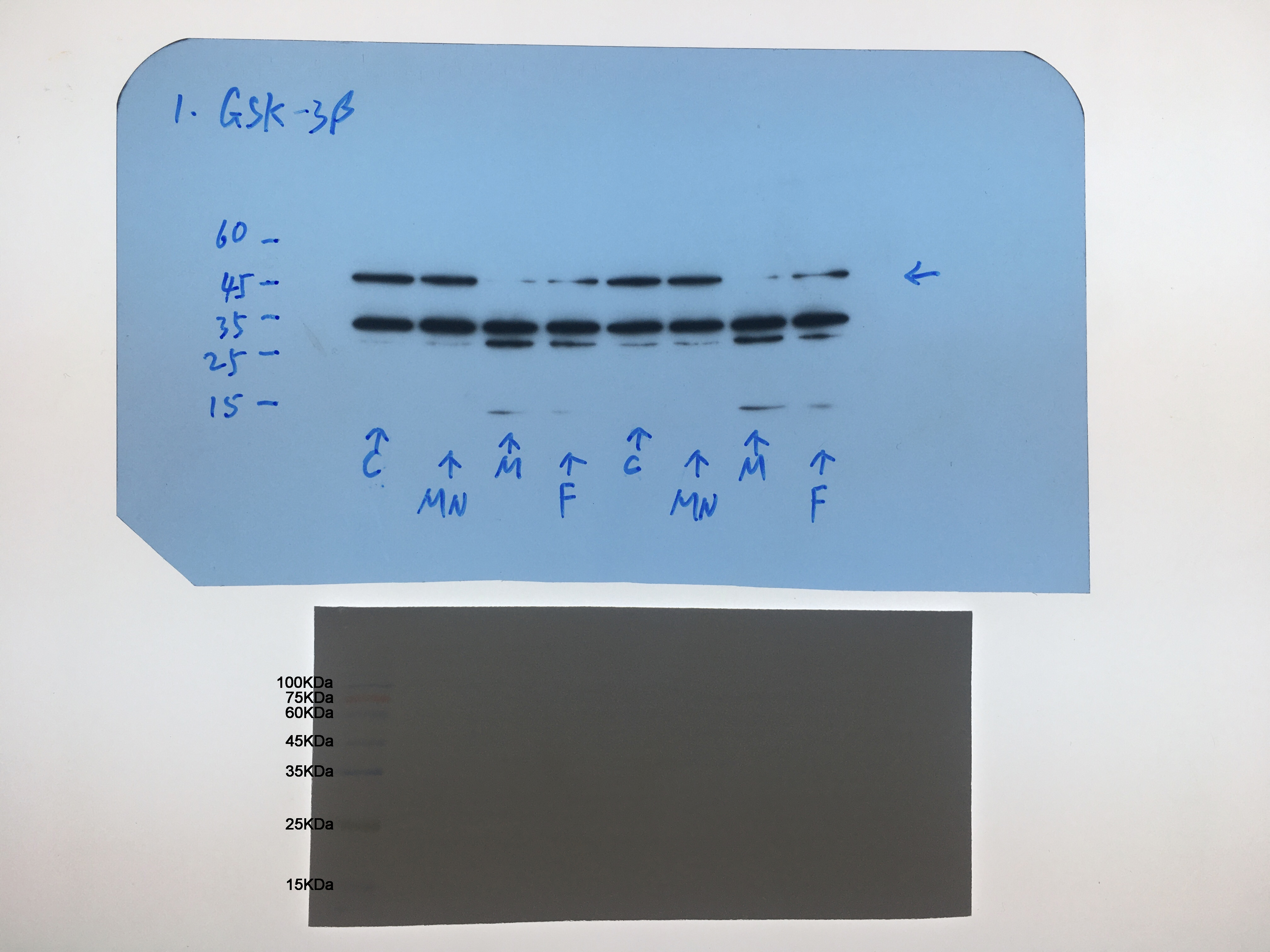

Supplement: Supplemental Information 3 [file peerj-11-14851-s003.zip › Figure6D-GSK-3β-1.jpg]

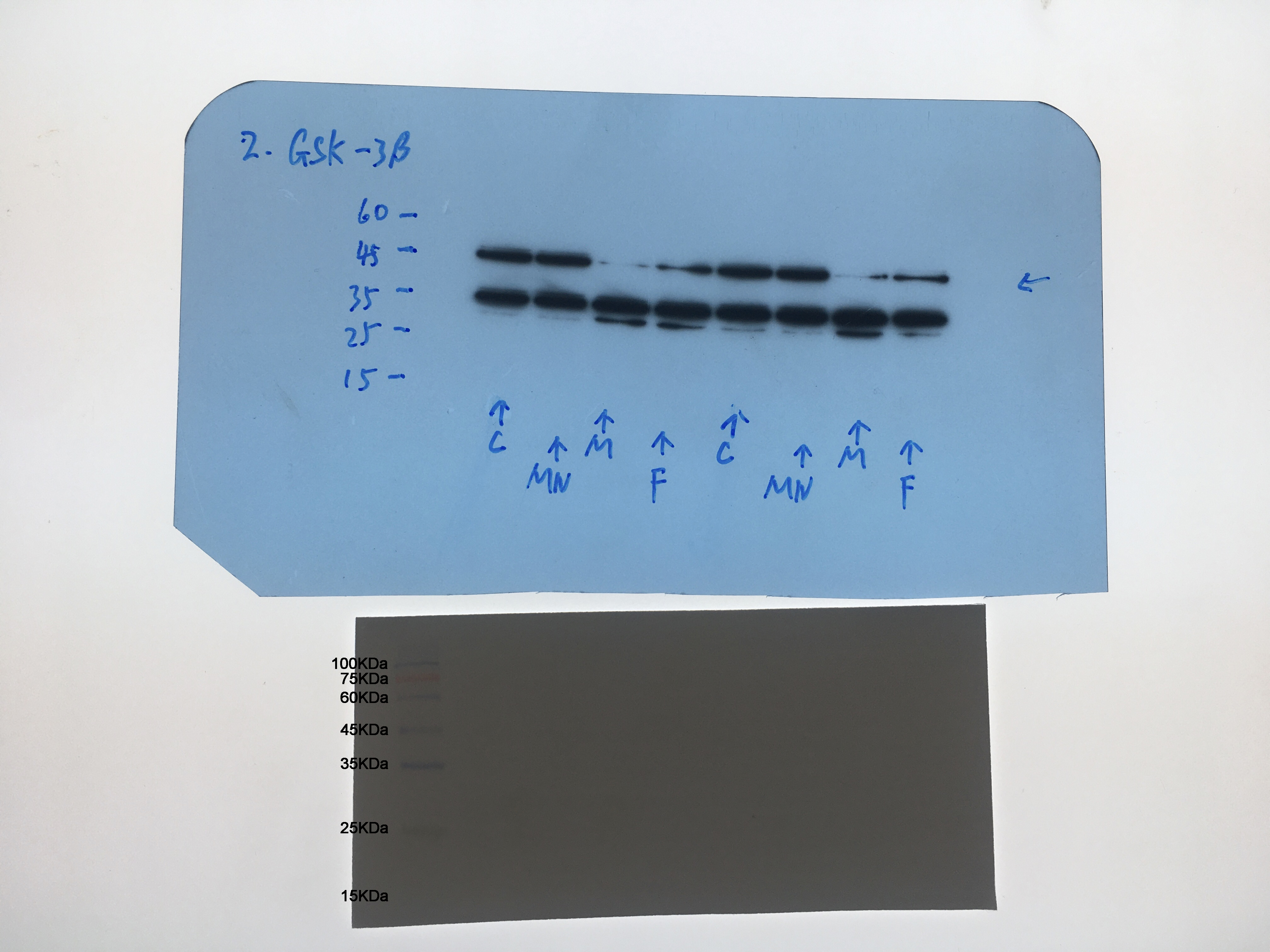

Supplement: Supplemental Information 3 [file peerj-11-14851-s003.zip › Figure6D-GSK-3β-2.jpg]
